# *cis*-(2,2′-Bipyridine-κ^2^ *N*,*N*′)bis­(isonicotinamide-κ*N* ^1^)palladium(II) bis­(hexa­fluoridophosphate)

**DOI:** 10.1107/S1600536812043036

**Published:** 2012-10-20

**Authors:** Rafael A. Adrian, Brayan Quintela, Douglas R. Powell, Seik Weng Ng, Edward R. T. Tiekink

**Affiliations:** a Department of Chemistry, University of the Incarnate Word, San Antonio, TX 78209, USA; b Department of Chemistry & Biochemistry, University of Oklahoma, Norman, OK 73019, USA; c Department of Chemistry, University of Malaya, 50603 Kuala Lumpur, Malaysia; d Chemistry Department, Faculty of Science, King Abdulaziz University, PO Box 80203 Jeddah, Saudi Arabia

## Abstract

In the title salt, [Pd(C_10_H_8_N_2_)(C_6_H_6_NO)_2_](PF_6_)_2_, the Pd^II^ atom is in a slightly distorted square-planar coordination environment by N atoms derived from two 4-pyridine­carboxamide ligands, in a *cis* disposition, and a chelating 2,2′-bipyridine mol­ecule. The monodentate ligands are nearly orthogonal to each other [dihedral angle = 85.7 (5)°] and to the PdN_4_ plane [dihedral angles = 79.3 (3) and 78.7 (3)°]. The amide O atoms lie to opposite sides of the PdN_4_ plane. The most notable feature of the crystal packing is a linear supra­molecular chain orientated approximately along [310] and formed *via* 16-membered {⋯HNCO}_4_ motifs. These are connected into a three-dimensional network by amide–H⋯O, F inter­actions. Both PF_6_^−^ anions are disordered over two positions of equal occupancy in respect of the F atoms.

## Related literature

For the synthesis of compounds with supra­molecular structures involving carboxamides as ligands, see: Sun *et al.* (2011 ▶); Moncol *et al.* (2007 ▶). For related palladium(II) complexes with isonicotinamide, see: Galstyan *et al.* (2011 ▶); Fujimura *et al.* (2004 ▶); Qin *et al.* (2001 ▶). For hydration of palladium-coordinated nitriles, see: Sanchez *et al.* (2000 ▶).
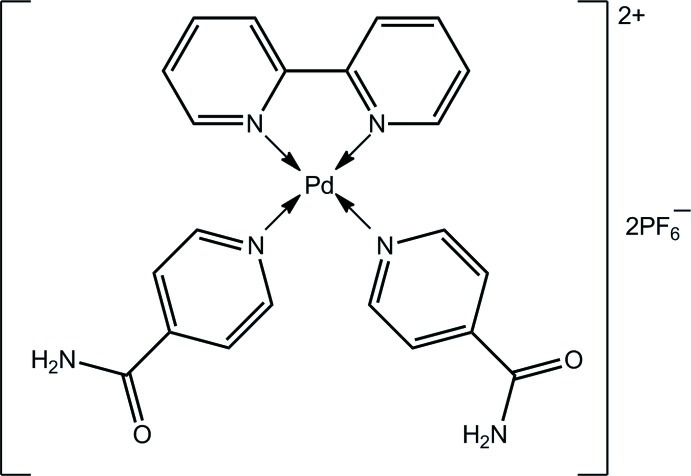


## Experimental

### 

#### Crystal data

[Pd(C_10_H_8_N_2_)(C_6_H_6_NO_2_)_2_](PF_6_)_2_
*M* *_r_* = 796.78
Monoclinic, 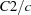

*a* = 22.5920 (14) Å
*b* = 13.8299 (8) Å
*c* = 17.9092 (12) Å
β = 95.269 (8)°
*V* = 5572.0 (6) Å^3^
*Z* = 8
Cu *K*α radiation
μ = 7.55 mm^−1^
*T* = 100 K
0.46 × 0.16 × 0.15 mm

#### Data collection

Bruker APEX CCD diffractometer
Absorption correction: multi-scan (*SADABS*; Sheldrick, 2007 ▶) *T*_min_ = 0.126, *T*_max_ = 0.401
30116 measured reflections
5182 independent reflections
3944 reflections with *I* > 2σ(*I*)
*R*_int_ = 0.072

#### Refinement

*R*[*F* ^2^ > 2σ(*F* ^2^)] = 0.052
*wR*(*F* ^2^) = 0.147
*S* = 1.01
5182 reflections
520 parameters
144 restraints
H-atom parameters constrained
Δρ_max_ = 0.67 e Å^−3^
Δρ_min_ = −0.78 e Å^−3^

### 

Data collection: *SMART* (Bruker, 1998 ▶); cell refinement: *SAINT* (Bruker, 1998 ▶); data reduction: *SAINT*; program(s) used to solve structure: *SHELXS97* (Sheldrick, 2008 ▶); program(s) used to refine structure: *SHELXL97* (Sheldrick, 2008 ▶); molecular graphics: *ORTEP-3 for Windows* (Farrugia, 2012 ▶) and *DIAMOND* (Brandenburg, 2006 ▶); software used to prepare material for publication: *publCIF* (Westrip, 2010 ▶).

## Supplementary Material

Crystal structure: contains datablock(s) global, I. DOI: 10.1107/S1600536812043036/su2513sup1.cif

Structure factors: contains datablock(s) I. DOI: 10.1107/S1600536812043036/su2513Isup2.hkl

Additional supplementary materials:  crystallographic information; 3D view; checkCIF report

## Figures and Tables

**Table 1:** Hydrogen-bond geometry (Å, °)

*D*—H⋯*A*	*D*—H	H⋯*A*	*D*⋯*A*	*D*—H⋯*A*
N2—H1N⋯O2^i^	0.88	2.29	3.031 (11)	142
N2—H1N⋯O2^ii^	0.88	2.37	3.032 (11)	132
N2—H2N⋯F9^iii^	0.88	2.24	3.037 (15)	151
N2—H2N⋯F11′^iii^	0.88	2.38	3.220 (14)	159
N4—H3N⋯F7′^iv^	0.88	2.52	3.175 (19)	132
N4—H3N⋯F8^iv^	0.88	2.55	3.10 (2)	121
N4—H4N⋯O1^v^	0.88	1.97	2.836 (11)	168

Symmetry codes: (i) 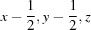; (ii) 
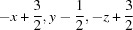
; (iii) 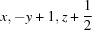; (iv) 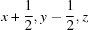; (v) 
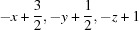
.

**Fig. 1.**
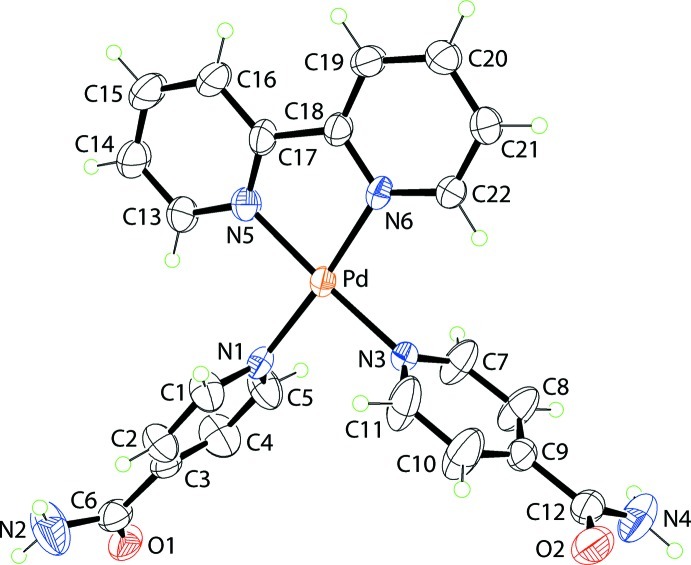
The molecular structure of the cation in the title salt showing the atom-labelling scheme and displacement ellipsoids at the 50% probability level.

**Fig. 2.**
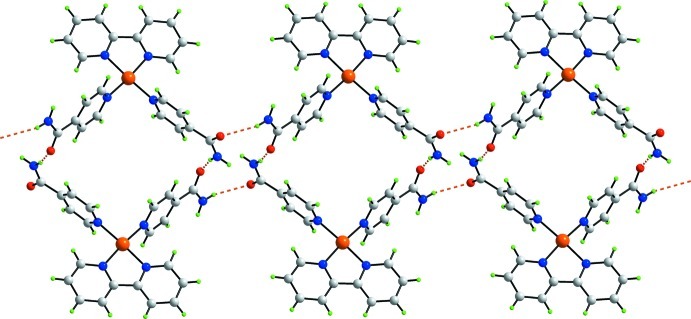
A view of the linear supramolecular chain mediated by N—H···O hydrogen bonds (orange dashed lines) along [13 5 -1] - see Table 1 for details.

## supplementary crystallographic information

### Comment

Carboxamides have been used for the synthesis of supramolecular structures due to their ability to hydrogen bond as shown by Sun *et al.* (2011) and Moncol *et al.* (2007). With that in mind we sought to crystallize palladium(II) complexes with 4-pyridinecarboxamide ligands, similar to those created by Galstyan *et al.*, (2011), Fujimura *et al.* (2004) and Qin *et al.* (2001). The title compound can be synthesized by the reaction of the 2,2'-bipyridine palladium(II) metal centre with the 4-pyridinecarboxamide ligand or by the palladium-catalyzed hydration of the 2,2'-bipyridine palladium(II) 4-cyanopyridine analog complex in a similar reaction to the one reported by Sanchez *et al.* (2000).

The complex cation of the title compound, Fig. 1, features a square planar palladium(II) centre with a *cis* disposition of the N-bound 4-pyridinecarboxamide ligands. No systematic trends are evident in the Pd—N bond lengths (Table 1). The 4-pyridinecarboxamide ligands are almost orthogonal to each other, the dihedral angle being 85.7 (5)°, and to the PdN_4_ plane with dihedral angles of 79.3 (3) [N1-ligand] and 78.7 (3)°; a similar situation was found in the BF_4_^-^ analogue (Qin *et al.*, 2001). Each of the amide groups lies out of the plane of the pyridyl ring to which it is connected as seen in the C2—C3—C6—O1 and C8—C9—C12—O2 torsion angles of -166.0 (8) and 171.5 (8)°, respectively. The 2,2'-bipyridine ring is almost planar the dihedral angle between the pyridyl rings being 4.7 (4)°. The amide-O atoms lie to opposite sides of the PdN_4_ plane.

A detailed analysis of the crystal packing is not possible owing to the disorder in the two PF_6_^-^ anions. However, the most notable feature of the crystal packing is a supramolecular chain orientated approximately along [3 1 0] arising from centrosymmetric 16-membered {···HNCO}_4_ synthons constructed by four interacting amide groups (Fig. 2 and Table 2). A three-dimensional architecture arises from additional amide-H···O, F interactions (Table 2).

### Experimental

Pd(bpy)(PF_6_)_2_ [bpy = 2,2'-bipyridine] was prepared by adding solid AgPF_6_ to an acetonitrile suspension of Pd(bpy)Cl_2_ (0.050 g, 0.15 mmol). After being stirred for 2 h, the mixture was filtered to remove AgCl. Isonicotinamide (0.037 g, 0.30 mmol) was added to the Pd(bpy)(PF_6_)_2_ solution and the solution was heated at 323 K for 3 h. Crystals were obtained by vapour diffusion of diethyl ether over an acetonitrile solution of the title complex [0.083 g, 85% yield; *M*.pt: 513 K (dec.), 523 K (melting)].

### Refinement

Ni and C-bound H-atoms were placed in calculated positions (N—H = 0.88 Å and C—H 0.95 Å) and were included in the refinement in the riding model approximation, with *U*_iso_(H) = 1.2*U*_equiv_(carrier atom). Both PF_6_ anions are disordered over two positions in respect of the F atoms only. The occupancy was assumed to be 1:1 as it could not be refined. The anisotropic displacement parameters of the F atoms were restrained to be nearly isotropic. Two reflections, *i.e*. (2 0 18) and (0 6 15), were omitted from the final refinement owing to poor agreement.

### Crystal data

**Table:**

[Pd(C_10_H_8_N_2_)(C_6_H_6_NO_2_)_2_](PF_6_)_2_	*F*(000) = 3152
*M**_r_* = 796.78	*D*_x_ = 1.900 Mg m^−^^3^
Monoclinic, *C*2/*c*	Cu *K*α radiation, λ = 1.54178 Å
Hall symbol: -C 2yc	Cell parameters from 4302 reflections
*a* = 22.5920 (14) Å	θ = 3.8–68.1°
*b* = 13.8299 (8) Å	µ = 7.55 mm^−^^1^
*c* = 17.9092 (12) Å	*T* = 100 K
β = 95.269 (8)°	Rod, colourless
*V* = 5572.0 (6) Å^3^	0.46 × 0.16 × 0.15 mm
*Z* = 8	

### Data collection

**Table:**

Bruker APEX CCD diffractometer	5182 independent reflections
Radiation source: fine-focus sealed tube	3944 reflections with *I* > 2σ(*I*)
Graphite monochromator	*R*_int_ = 0.072
φ and ω scans	θ_max_ = 69.8°, θ_min_ = 3.8°
Absorption correction: multi-scan (*SADABS*; Sheldrick, 2007)	*h* = −26→26
*T*_min_ = 0.126, *T*_max_ = 0.401	*k* = −16→16
30116 measured reflections	*l* = −21→18

### Refinement

**Table:**

Refinement on *F*^2^	Primary atom site location: structure-invariant direct methods
Least-squares matrix: full	Secondary atom site location: difference Fourier map
*R*[*F*^2^ > 2σ(*F*^2^)] = 0.052	Hydrogen site location: inferred from neighbouring sites
*wR*(*F*^2^) = 0.147	H-atom parameters constrained
*S* = 1.01	*w* = 1/[σ^2^(*F*_o_^2^) + (0.0879*P*)^2^ + 9.8993*P*] where *P* = (*F*_o_^2^ + 2*F*_c_^2^)/3
5182 reflections	(Δ/σ)_max_ = 0.001
520 parameters	Δρ_max_ = 0.67 e Å^−^^3^
144 restraints	Δρ_min_ = −0.78 e Å^−^^3^

### Special details

**Table:**

Refinement. Refinement of *F*^2^ against ALL reflections. The weighted *R*-factor *wR* and goodness of fit *S* are based on *F*^2^, conventional *R*-factors *R* are based on *F*, with *F* set to zero for negative *F*^2^. The threshold expression of *F*^2^ > σ(*F*^2^) is used only for calculating *R*-factors(gt) *etc*. and is not relevant to the choice of reflections for refinement. *R*-factors based on *F*^2^ are statistically about twice as large as those based on *F*, and *R*- factors based on ALL data will be even larger.

### Fractional atomic coordinates and isotropic or equivalent isotropic displacement parameters (Å^2^)

**Table:**

	*x*	*y*	*z*	*U*_iso_*/*U*_eq_	Occ. (<1)
Pd	0.667091 (18)	0.53803 (3)	0.62000 (2)	0.03743 (15)	
P1	0.70718 (7)	0.64260 (15)	0.84670 (11)	0.0532 (4)	
P2	0.57543 (8)	0.73478 (13)	0.41320 (11)	0.0519 (4)	
F1	0.7509 (5)	0.6745 (11)	0.7858 (7)	0.086 (3)	0.50
F2	0.7240 (7)	0.7410 (9)	0.8898 (9)	0.118 (4)	0.50
F3	0.6661 (5)	0.6128 (12)	0.9038 (7)	0.093 (4)	0.50
F4	0.6999 (6)	0.5423 (8)	0.8021 (6)	0.084 (3)	0.50
F5	0.6557 (5)	0.6892 (10)	0.7948 (8)	0.087 (4)	0.50
F6	0.7629 (4)	0.5972 (6)	0.8934 (6)	0.052 (2)	0.50
F7	0.5623 (7)	0.6485 (8)	0.4674 (6)	0.088 (3)	0.50
F8	0.5449 (7)	0.8077 (15)	0.4605 (11)	0.081 (5)	0.50
F9	0.5970 (6)	0.8181 (7)	0.3611 (7)	0.077 (3)	0.50
F10	0.6113 (4)	0.6573 (8)	0.3638 (6)	0.052 (2)	0.50
F11	0.6403 (4)	0.7503 (9)	0.4640 (6)	0.078 (3)	0.50
F12	0.5198 (4)	0.7132 (10)	0.3636 (7)	0.087 (3)	0.50
F1'	0.7399 (10)	0.5757 (17)	0.7956 (11)	0.160 (7)	0.50
F2'	0.7296 (7)	0.7305 (12)	0.8075 (9)	0.118 (5)	0.50
F3'	0.6705 (6)	0.7100 (9)	0.8982 (6)	0.089 (3)	0.50
F4'	0.6788 (7)	0.5562 (9)	0.8956 (8)	0.095 (4)	0.50
F5'	0.6495 (6)	0.6323 (10)	0.7903 (7)	0.082 (3)	0.50
F6'	0.7612 (9)	0.6409 (15)	0.9077 (11)	0.136 (7)	0.50
F7'	0.5639 (7)	0.8201 (13)	0.4737 (11)	0.065 (4)	0.50
F8'	0.6250 (4)	0.7947 (9)	0.3815 (7)	0.066 (3)	0.50
F9'	0.5184 (6)	0.6781 (9)	0.4411 (7)	0.094 (3)	0.50
F10'	0.5814 (6)	0.6529 (10)	0.3528 (7)	0.081 (3)	0.50
F11'	0.5277 (4)	0.7880 (9)	0.3518 (6)	0.081 (3)	0.50
F12'	0.6163 (6)	0.6839 (10)	0.4707 (6)	0.094 (3)	0.50
O1	0.5848 (2)	0.0479 (3)	0.5984 (4)	0.0706 (16)	
O2	0.9674 (2)	0.4298 (4)	0.6755 (4)	0.0730 (16)	
N1	0.6458 (2)	0.3976 (4)	0.6254 (3)	0.0426 (11)	
N2	0.5616 (5)	0.0829 (6)	0.7123 (5)	0.105 (3)	
H1n	0.5465	0.0249	0.7176	0.126*	
H2n	0.5616	0.1254	0.7488	0.126*	
N3	0.7528 (2)	0.4988 (4)	0.6136 (3)	0.0393 (11)	
N4	0.9639 (3)	0.4321 (6)	0.5525 (5)	0.086 (2)	
H3n	1.0022	0.4192	0.5553	0.103*	
H4n	0.9437	0.4393	0.5085	0.103*	
N5	0.5835 (2)	0.5845 (4)	0.6278 (3)	0.0459 (12)	
N6	0.6826 (2)	0.6815 (4)	0.6218 (3)	0.0407 (11)	
C1	0.6372 (4)	0.3601 (5)	0.6929 (4)	0.0602 (19)	
H1	0.6435	0.3999	0.7361	0.072*	
C2	0.6201 (4)	0.2674 (5)	0.7010 (4)	0.0586 (18)	
H2	0.6160	0.2428	0.7498	0.070*	
C3	0.6084 (3)	0.2081 (4)	0.6399 (4)	0.0455 (15)	
C4	0.6180 (5)	0.2470 (6)	0.5720 (5)	0.077 (3)	
H4	0.6113	0.2087	0.5280	0.092*	
C5	0.6369 (4)	0.3400 (6)	0.5663 (5)	0.068 (2)	
H5	0.6440	0.3644	0.5184	0.082*	
C6	0.5854 (3)	0.1070 (5)	0.6467 (5)	0.0577 (18)	
C7	0.7775 (3)	0.5003 (8)	0.5506 (5)	0.071 (2)	
H7	0.7535	0.5146	0.5055	0.085*	
C8	0.8376 (4)	0.4816 (8)	0.5470 (5)	0.074 (3)	
H8	0.8545	0.4856	0.5005	0.089*	
C9	0.8717 (3)	0.4578 (4)	0.6095 (4)	0.0468 (15)	
C10	0.8457 (4)	0.4501 (9)	0.6735 (6)	0.097 (4)	
H10	0.8684	0.4304	0.7182	0.116*	
C11	0.7862 (4)	0.4707 (9)	0.6746 (5)	0.090 (3)	
H11	0.7684	0.4646	0.7205	0.108*	
C12	0.9376 (3)	0.4405 (5)	0.6127 (5)	0.0546 (18)	
C13	0.5341 (3)	0.5284 (5)	0.6259 (5)	0.0560 (17)	
H13	0.5372	0.4608	0.6179	0.067*	
C14	0.4796 (3)	0.5682 (6)	0.6354 (5)	0.068 (2)	
H14	0.4451	0.5286	0.6333	0.081*	
C15	0.4755 (3)	0.6653 (6)	0.6479 (6)	0.082 (3)	
H15	0.4384	0.6936	0.6560	0.098*	
C16	0.5259 (3)	0.7221 (6)	0.6486 (5)	0.068 (2)	
H16	0.5236	0.7898	0.6569	0.082*	
C17	0.5794 (3)	0.6798 (5)	0.6372 (4)	0.0497 (16)	
C18	0.6345 (3)	0.7349 (5)	0.6329 (4)	0.0435 (14)	
C19	0.6381 (3)	0.8338 (5)	0.6376 (4)	0.0520 (16)	
H19	0.6037	0.8706	0.6452	0.062*	
C20	0.6913 (3)	0.8800 (5)	0.6313 (4)	0.0558 (17)	
H20	0.6939	0.9485	0.6336	0.067*	
C21	0.7415 (3)	0.8240 (5)	0.6214 (4)	0.0545 (17)	
H21	0.7791	0.8536	0.6178	0.065*	
C22	0.7353 (3)	0.7249 (5)	0.6170 (4)	0.0437 (14)	
H22	0.7692	0.6863	0.6104	0.052*	

### Atomic displacement parameters (Å^2^)

**Table:**

	*U*^11^	*U*^22^	*U*^33^	*U*^12^	*U*^13^	*U*^23^
Pd	0.0313 (2)	0.0397 (2)	0.0422 (3)	−0.00218 (17)	0.00854 (15)	0.00002 (19)
P1	0.0333 (8)	0.0718 (11)	0.0553 (11)	0.0101 (7)	0.0084 (7)	0.0075 (9)
P2	0.0489 (10)	0.0513 (9)	0.0566 (11)	−0.0045 (7)	0.0104 (7)	−0.0003 (8)
F1	0.052 (5)	0.120 (8)	0.085 (7)	−0.023 (5)	0.009 (4)	0.023 (6)
F2	0.135 (8)	0.078 (6)	0.133 (9)	0.028 (6)	−0.024 (7)	−0.028 (6)
F3	0.074 (6)	0.141 (9)	0.069 (6)	0.002 (6)	0.034 (5)	0.002 (7)
F4	0.096 (7)	0.077 (6)	0.076 (6)	−0.036 (5)	−0.006 (5)	−0.024 (5)
F5	0.057 (6)	0.116 (8)	0.085 (7)	0.031 (6)	−0.007 (5)	0.019 (7)
F6	0.041 (4)	0.042 (4)	0.069 (5)	0.010 (3)	−0.005 (3)	0.002 (4)
F7	0.118 (8)	0.082 (6)	0.067 (6)	−0.025 (6)	0.027 (6)	0.011 (5)
F8	0.078 (9)	0.091 (8)	0.078 (8)	0.009 (7)	0.023 (7)	−0.031 (6)
F9	0.119 (8)	0.039 (4)	0.074 (6)	−0.011 (5)	0.022 (6)	0.000 (4)
F10	0.044 (4)	0.055 (5)	0.057 (5)	0.008 (4)	0.000 (4)	−0.001 (4)
F11	0.057 (5)	0.100 (7)	0.076 (6)	−0.001 (5)	−0.006 (4)	−0.021 (5)
F12	0.048 (5)	0.112 (7)	0.098 (7)	0.010 (5)	−0.003 (5)	−0.015 (6)
F1'	0.158 (10)	0.174 (11)	0.155 (10)	0.065 (9)	0.050 (8)	−0.009 (8)
F2'	0.122 (9)	0.118 (8)	0.115 (9)	−0.037 (7)	0.016 (7)	0.042 (7)
F3'	0.118 (7)	0.084 (6)	0.068 (6)	0.025 (6)	0.020 (5)	−0.010 (5)
F4'	0.122 (8)	0.068 (6)	0.096 (8)	−0.010 (6)	0.008 (6)	0.018 (6)
F5'	0.075 (6)	0.104 (7)	0.066 (6)	−0.011 (6)	0.001 (5)	0.001 (6)
F6'	0.098 (9)	0.172 (11)	0.134 (10)	−0.014 (9)	−0.002 (7)	0.031 (9)
F7'	0.063 (7)	0.063 (6)	0.071 (7)	−0.003 (5)	0.016 (6)	−0.014 (5)
F8'	0.045 (5)	0.081 (6)	0.075 (6)	−0.013 (4)	0.019 (4)	−0.019 (5)
F9'	0.091 (7)	0.097 (7)	0.095 (7)	−0.034 (6)	0.018 (6)	0.012 (6)
F10'	0.118 (8)	0.064 (5)	0.063 (6)	−0.011 (7)	0.012 (7)	−0.002 (5)
F11'	0.049 (5)	0.109 (7)	0.083 (6)	0.015 (5)	0.002 (4)	0.028 (5)
F12'	0.101 (7)	0.107 (7)	0.073 (6)	0.040 (6)	0.001 (5)	0.009 (6)
O1	0.058 (3)	0.043 (3)	0.115 (5)	−0.003 (2)	0.031 (3)	−0.020 (3)
O2	0.049 (3)	0.063 (3)	0.106 (5)	0.008 (2)	0.002 (3)	−0.007 (3)
N1	0.031 (2)	0.051 (3)	0.047 (3)	0.002 (2)	0.012 (2)	−0.004 (2)
N2	0.158 (9)	0.066 (5)	0.101 (7)	−0.041 (5)	0.058 (6)	−0.016 (4)
N3	0.035 (2)	0.038 (2)	0.046 (3)	0.0011 (19)	0.011 (2)	0.001 (2)
N4	0.051 (4)	0.109 (6)	0.101 (6)	0.006 (4)	0.025 (4)	−0.022 (5)
N5	0.037 (3)	0.047 (3)	0.053 (3)	−0.006 (2)	0.002 (2)	−0.001 (2)
N6	0.025 (2)	0.050 (3)	0.047 (3)	−0.0016 (19)	0.0054 (19)	0.000 (2)
C1	0.077 (5)	0.053 (4)	0.052 (5)	−0.016 (4)	0.018 (4)	−0.011 (3)
C2	0.073 (5)	0.048 (4)	0.055 (5)	−0.013 (3)	0.007 (3)	0.001 (3)
C3	0.037 (3)	0.033 (3)	0.066 (5)	0.004 (2)	0.007 (3)	−0.006 (3)
C4	0.114 (8)	0.058 (4)	0.061 (6)	−0.015 (5)	0.019 (5)	−0.023 (4)
C5	0.099 (6)	0.060 (4)	0.049 (5)	−0.024 (4)	0.022 (4)	−0.008 (3)
C6	0.051 (4)	0.048 (4)	0.075 (5)	0.004 (3)	0.010 (3)	−0.009 (4)
C7	0.045 (4)	0.122 (7)	0.047 (5)	0.019 (4)	0.013 (3)	−0.005 (4)
C8	0.053 (4)	0.123 (8)	0.049 (5)	0.027 (5)	0.017 (3)	−0.008 (4)
C9	0.038 (3)	0.037 (3)	0.066 (4)	−0.005 (2)	0.009 (3)	−0.009 (3)
C10	0.047 (5)	0.170 (12)	0.074 (6)	0.026 (6)	0.010 (4)	0.037 (7)
C11	0.047 (5)	0.167 (11)	0.059 (6)	0.007 (5)	0.018 (4)	0.035 (6)
C12	0.044 (4)	0.042 (3)	0.078 (5)	−0.001 (3)	0.009 (3)	−0.010 (3)
C13	0.036 (3)	0.056 (4)	0.075 (5)	−0.008 (3)	0.001 (3)	0.005 (3)
C14	0.040 (4)	0.066 (5)	0.098 (7)	−0.006 (3)	0.010 (4)	−0.004 (4)
C15	0.039 (4)	0.069 (5)	0.139 (9)	0.006 (4)	0.020 (4)	−0.005 (5)
C16	0.038 (4)	0.059 (4)	0.111 (7)	0.007 (3)	0.016 (4)	0.000 (4)
C17	0.032 (3)	0.058 (4)	0.060 (4)	−0.004 (3)	0.009 (3)	0.006 (3)
C18	0.033 (3)	0.047 (3)	0.051 (4)	−0.003 (2)	0.006 (2)	−0.002 (3)
C19	0.041 (3)	0.042 (3)	0.073 (5)	0.003 (3)	0.004 (3)	−0.008 (3)
C20	0.050 (4)	0.042 (3)	0.075 (5)	−0.003 (3)	0.004 (3)	−0.005 (3)
C21	0.043 (4)	0.049 (4)	0.071 (5)	−0.010 (3)	0.005 (3)	−0.003 (3)
C22	0.034 (3)	0.045 (3)	0.052 (4)	0.000 (2)	0.003 (2)	0.002 (3)

### Geometric parameters (Å, º)

**Table:**

Pd—N1	2.005 (5)	N5—C17	1.332 (9)
Pd—N3	2.024 (5)	N5—C13	1.357 (8)
Pd—N5	2.013 (5)	N6—C22	1.343 (7)
Pd—N6	2.014 (5)	N6—C18	1.346 (8)
P1—F3	1.501 (11)	C1—C2	1.351 (10)
P1—F2'	1.514 (13)	C1—H1	0.9500
P1—F1'	1.538 (16)	C2—C3	1.374 (10)
P1—F5	1.561 (11)	C2—H2	0.9500
P1—F6'	1.562 (19)	C3—C4	1.365 (11)
P1—F6	1.577 (9)	C3—C6	1.501 (9)
P1—F5'	1.580 (13)	C4—C5	1.362 (11)
P1—F2	1.593 (13)	C4—H4	0.9500
P1—F3'	1.596 (10)	C5—H5	0.9500
P1—F1	1.599 (11)	C7—C8	1.391 (10)
P1—F4	1.602 (10)	C7—H7	0.9500
P1—F4'	1.646 (12)	C8—C9	1.340 (11)
P2—F12'	1.494 (11)	C8—H8	0.9500
P2—F12	1.500 (10)	C9—C10	1.339 (12)
P2—F8	1.522 (19)	C9—C12	1.504 (9)
P2—F8'	1.542 (11)	C10—C11	1.376 (12)
P2—F10'	1.580 (13)	C10—H10	0.9500
P2—F7	1.583 (10)	C11—H11	0.9500
P2—F9	1.587 (11)	C13—C14	1.373 (10)
P2—F9'	1.626 (11)	C13—H13	0.9500
P2—F7'	1.639 (19)	C14—C15	1.366 (12)
P2—F11'	1.642 (10)	C14—H14	0.9500
P2—F10	1.650 (11)	C15—C16	1.382 (11)
P2—F11	1.667 (10)	C15—H15	0.9500
O1—C6	1.189 (9)	C16—C17	1.374 (9)
O2—C12	1.266 (10)	C16—H16	0.9500
N1—C5	1.325 (9)	C17—C18	1.467 (8)
N1—C1	1.345 (9)	C18—C19	1.372 (9)
N2—C6	1.376 (11)	C19—C20	1.375 (9)
N2—H1n	0.8800	C19—H19	0.9500
N2—H2n	0.8800	C20—C21	1.398 (10)
N3—C7	1.303 (9)	C20—H20	0.9500
N3—C11	1.329 (10)	C21—C22	1.380 (9)
N4—C12	1.282 (10)	C21—H21	0.9500
N4—H3n	0.8800	C22—H22	0.9500
N4—H4n	0.8800		
			
N1—Pd—N5	94.3 (2)	C11—N3—Pd	120.4 (5)
N1—Pd—N6	174.40 (19)	C12—N4—H3n	120.0
N5—Pd—N6	81.2 (2)	C12—N4—H4n	120.0
N1—Pd—N3	88.8 (2)	H3n—N4—H4n	120.0
N5—Pd—N3	176.8 (2)	C17—N5—C13	120.2 (6)
N6—Pd—N3	95.66 (19)	C17—N5—Pd	113.6 (4)
F2'—P1—F1'	90.4 (10)	C13—N5—Pd	126.2 (5)
F3—P1—F5	92.6 (8)	C22—N6—C18	119.8 (6)
F3—P1—F6'	90.7 (9)	C22—N6—Pd	126.4 (4)
F2'—P1—F6'	93.6 (10)	C18—N6—Pd	113.7 (4)
F1'—P1—F6'	91.1 (11)	N1—C1—C2	121.9 (7)
F5—P1—F6'	156.2 (10)	N1—C1—H1	119.0
F3—P1—F6	92.5 (7)	C2—C1—H1	119.0
F5—P1—F6	174.9 (7)	C1—C2—C3	121.1 (7)
F2'—P1—F5'	93.9 (8)	C1—C2—H2	119.4
F1'—P1—F5'	88.8 (10)	C3—C2—H2	119.4
F6'—P1—F5'	172.5 (9)	C4—C3—C2	116.1 (6)
F3—P1—F2	92.3 (9)	C4—C3—C6	121.7 (7)
F5—P1—F2	93.9 (8)	C2—C3—C6	122.2 (7)
F6—P1—F2	86.6 (6)	C5—C4—C3	121.0 (7)
F2'—P1—F3'	90.8 (9)	C5—C4—H4	119.5
F1'—P1—F3'	177.4 (11)	C3—C4—H4	119.5
F6'—P1—F3'	91.1 (9)	N1—C5—C4	122.3 (8)
F5'—P1—F3'	88.9 (7)	N1—C5—H5	118.9
F3—P1—F1	179.9 (10)	C4—C5—H5	118.9
F5—P1—F1	87.4 (7)	O1—C6—N2	118.3 (7)
F6—P1—F1	87.6 (6)	O1—C6—C3	124.2 (7)
F2—P1—F1	87.7 (8)	N2—C6—C3	117.4 (7)
F3—P1—F4	93.5 (8)	N3—C7—C8	122.3 (8)
F5—P1—F4	91.3 (7)	N3—C7—H7	118.9
F6—P1—F4	87.8 (6)	C8—C7—H7	118.9
F2—P1—F4	172.1 (8)	C9—C8—C7	119.8 (7)
F1—P1—F4	86.6 (7)	C9—C8—H8	120.1
F2'—P1—F4'	173.2 (9)	C7—C8—H8	120.1
F1'—P1—F4'	96.4 (11)	C10—C9—C8	118.1 (7)
F6'—P1—F4'	86.3 (9)	C10—C9—C12	117.8 (7)
F5'—P1—F4'	86.3 (7)	C8—C9—C12	124.1 (7)
F3'—P1—F4'	82.4 (7)	C9—C10—C11	120.1 (8)
F12—P2—F8	93.7 (8)	C9—C10—H10	120.0
F12'—P2—F8'	94.8 (8)	C11—C10—H10	120.0
F12'—P2—F10'	92.7 (7)	N3—C11—C10	122.1 (8)
F8'—P2—F10'	91.3 (6)	N3—C11—H11	119.0
F12—P2—F7	91.1 (7)	C10—C11—H11	119.0
F8—P2—F7	92.2 (10)	O2—C12—N4	119.1 (8)
F12—P2—F9	94.7 (7)	O2—C12—C9	119.7 (7)
F8—P2—F9	91.4 (9)	N4—C12—C9	121.1 (8)
F10'—P2—F9	93.9 (6)	N5—C13—C14	120.8 (7)
F7—P2—F9	172.9 (8)	N5—C13—H13	119.6
F12'—P2—F9'	91.1 (8)	C14—C13—H13	119.6
F8'—P2—F9'	174.1 (7)	C15—C14—C13	119.2 (7)
F10'—P2—F9'	89.1 (7)	C15—C14—H14	120.4
F12'—P2—F7'	90.6 (8)	C13—C14—H14	120.4
F8'—P2—F7'	91.2 (7)	C14—C15—C16	119.5 (7)
F10'—P2—F7'	175.7 (8)	C14—C15—H15	120.2
F9'—P2—F7'	88.0 (7)	C16—C15—H15	120.2
F12'—P2—F11'	177.2 (7)	C17—C16—C15	119.4 (8)
F8'—P2—F11'	88.1 (6)	C17—C16—H16	120.3
F10'—P2—F11'	87.4 (7)	C15—C16—H16	120.3
F9'—P2—F11'	86.0 (6)	N5—C17—C16	120.8 (6)
F7'—P2—F11'	89.2 (8)	N5—C17—C18	116.0 (6)
F12—P2—F10	88.8 (5)	C16—C17—C18	123.2 (7)
F8—P2—F10	177.5 (7)	N6—C18—C19	120.8 (6)
F7—P2—F10	88.1 (6)	N6—C18—C17	115.1 (6)
F9—P2—F10	88.0 (6)	C19—C18—C17	124.1 (6)
F12—P2—F11	174.5 (6)	C18—C19—C20	120.4 (6)
F8—P2—F11	91.8 (7)	C18—C19—H19	119.8
F7—P2—F11	88.1 (7)	C20—C19—H19	119.8
F9—P2—F11	85.7 (7)	C19—C20—C21	118.6 (6)
F10—P2—F11	85.8 (5)	C19—C20—H20	120.7
C5—N1—C1	117.5 (6)	C21—C20—H20	120.7
C5—N1—Pd	124.3 (5)	C22—C21—C20	118.6 (6)
C1—N1—Pd	118.2 (4)	C22—C21—H21	120.7
C6—N2—H1n	120.0	C20—C21—H21	120.7
C6—N2—H2n	120.0	N6—C22—C21	121.8 (6)
H1n—N2—H2n	120.0	N6—C22—H22	119.1
C7—N3—C11	117.4 (6)	C21—C22—H22	119.1
C7—N3—Pd	122.2 (5)		
			
N5—Pd—N1—C5	99.2 (6)	C8—C9—C10—C11	−2.9 (16)
N3—Pd—N1—C5	−81.7 (6)	C12—C9—C10—C11	176.3 (10)
N5—Pd—N1—C1	−77.7 (5)	C7—N3—C11—C10	4.4 (16)
N3—Pd—N1—C1	101.4 (5)	Pd—N3—C11—C10	−175.3 (9)
N1—Pd—N3—C7	103.1 (7)	C9—C10—C11—N3	−0.2 (19)
N6—Pd—N3—C7	−80.3 (7)	C10—C9—C12—O2	−7.7 (11)
N1—Pd—N3—C11	−77.2 (7)	C8—C9—C12—O2	171.5 (8)
N6—Pd—N3—C11	99.4 (7)	C10—C9—C12—N4	169.4 (9)
N1—Pd—N5—C17	170.8 (5)	C8—C9—C12—N4	−11.5 (12)
N6—Pd—N5—C17	−5.9 (5)	C17—N5—C13—C14	−1.6 (12)
N1—Pd—N5—C13	−7.8 (6)	Pd—N5—C13—C14	176.9 (6)
N6—Pd—N5—C13	175.6 (6)	N5—C13—C14—C15	−0.9 (14)
N5—Pd—N6—C22	−178.6 (6)	C13—C14—C15—C16	1.8 (16)
N3—Pd—N6—C22	2.1 (6)	C14—C15—C16—C17	−0.4 (15)
N5—Pd—N6—C18	4.9 (5)	C13—N5—C17—C16	3.0 (11)
N3—Pd—N6—C18	−174.4 (5)	Pd—N5—C17—C16	−175.6 (6)
C5—N1—C1—C2	−0.3 (12)	C13—N5—C17—C18	−175.6 (6)
Pd—N1—C1—C2	176.9 (6)	Pd—N5—C17—C18	5.8 (8)
N1—C1—C2—C3	−2.4 (13)	C15—C16—C17—N5	−2.0 (13)
C1—C2—C3—C4	3.0 (12)	C15—C16—C17—C18	176.5 (8)
C1—C2—C3—C6	−175.9 (7)	C22—N6—C18—C19	1.5 (10)
C2—C3—C4—C5	−1.2 (13)	Pd—N6—C18—C19	178.2 (5)
C6—C3—C4—C5	177.7 (8)	C22—N6—C18—C17	−180.0 (6)
C1—N1—C5—C4	2.1 (13)	Pd—N6—C18—C17	−3.3 (7)
Pd—N1—C5—C4	−174.8 (8)	N5—C17—C18—N6	−1.7 (9)
C3—C4—C5—N1	−1.4 (16)	C16—C17—C18—N6	179.7 (7)
C4—C3—C6—O1	15.2 (12)	N5—C17—C18—C19	176.7 (7)
C2—C3—C6—O1	−166.0 (8)	C16—C17—C18—C19	−1.8 (12)
C4—C3—C6—N2	−162.3 (9)	N6—C18—C19—C20	−0.2 (11)
C2—C3—C6—N2	16.6 (11)	C17—C18—C19—C20	−178.6 (7)
C11—N3—C7—C8	−5.4 (14)	C18—C19—C20—C21	−1.1 (12)
Pd—N3—C7—C8	174.2 (8)	C19—C20—C21—C22	1.1 (12)
N3—C7—C8—C9	2.4 (16)	C18—N6—C22—C21	−1.5 (10)
C7—C8—C9—C10	2.0 (14)	Pd—N6—C22—C21	−177.7 (5)
C7—C8—C9—C12	−177.2 (8)	C20—C21—C22—N6	0.1 (11)

### Hydrogen-bond geometry (Å, º)

**Table:**

*D*—H···*A*	*D*—H	H···*A*	*D*···*A*	*D*—H···*A*
N2—H1N···O2^i^	0.88	2.29	3.031 (11)	142
N2—H1N···O2^ii^	0.88	2.37	3.032 (11)	132
N2—H2N···F9^iii^	0.88	2.24	3.037 (15)	151
N2—H2N···F11′^iii^	0.88	2.38	3.220 (14)	159
N4—H3N···F7′^iv^	0.88	2.52	3.175 (19)	132
N4—H3N···F8^iv^	0.88	2.55	3.10 (2)	121
N4—H4N···O1^v^	0.88	1.97	2.836 (11)	168

Symmetry codes: (i) *x*−1/2, *y*−1/2, *z*; (ii) −*x*+3/2, *y*−1/2, −*z*+3/2; (iii) *x*, −*y*+1, *z*+1/2; (iv) *x*+1/2, *y*−1/2, *z*; (v) −*x*+3/2, −*y*+1/2, −*z*+1.
